# Neuropathology changed by 3- and 6-months low-level PM_2.5_ inhalation exposure in spontaneously hypertensive rats

**DOI:** 10.1186/s12989-020-00388-6

**Published:** 2020-11-26

**Authors:** Hsiao-Chi Chuang, Hsin-Chang Chen, Pei-Jui Chai, Ho-Tang Liao, Chang-Fu Wu, Chia-Ling Chen, Ming-Kai Jhan, Hui-I Hsieh, Kuen-Yuh Wu, Ta-Fu Chen, Tsun-Jen Cheng

**Affiliations:** 1 School of Respiratory Therapy, College of Medicine, Taipei Medical University, Taipei, Taiwan; 2 Division of Pulmonary Medicine, Department of Internal Medicine, Shuang Ho Hospital, Taipei Medical University, New Taipei City, Taiwan; 3 Cell Physiology and Molecular Image Research Center, Wan Fang Hospital, Taipei Medical University, Taipei, Taiwan; 4 Institute of Food Safety and Health, College of Public Health, National Taiwan University, Taipei, Taiwan; 5 Institute of Environmental and Occupational Health Science, College of Public Health, National Taiwan University, 17 Xu-Zhou Road, Taipei, 100 Taiwan; 6 Department of Public Health, College of Public Health, National Taiwan University, Taipei, Taiwan; 7 Graduate Institute of Medical Sciences, College of Medicine, Taipei Medical University, Taipei, Taiwan; 8 Department of Microbiology and Immunology, School of Medicine, College of Medicine, Taipei Medical University, Taipei, Taiwan; 9 Department of Occupational Medicine, Cathay General Hospital, Taipei, Taiwan; 10 Department of Neurology, National Taiwan University Hospital, College of Medicine, National Taiwan University, No. 1, Changde Street, Taipei, 10048 Taiwan

**Keywords:** Air pollution, Autophagy, Central nervous system toxicity, Particulate matter, Tau

## Abstract

**Background:**

Epidemiological evidence has linked fine particulate matter (PM_2.5_) to neurodegenerative diseases; however, the toxicological evidence remains unclear. The objective of this study was to investigate the effects of PM_2.5_ on neuropathophysiology in a hypertensive animal model. We examined behavioral alterations (Morris water maze), lipid peroxidation (malondialdehyde (MDA)), tau and autophagy expressions, neuron death, and caspase-3 levels after 3 and 6 months of whole-body exposure to urban PM_2.5_ in spontaneously hypertensive (SH) rats.

**Results:**

SH rats were exposed to S-, K-, Si-, and Fe-dominated PM_2.5_ at 8.6 ± 2.5 and 10.8 ± 3.8 μg/m^3^ for 3 and 6 months, respectively. We observed no significant alterations in the escape latency, distance moved, mean area crossing, mean time spent, or mean swimming velocity after PM_2.5_ exposure. Notably, levels of MDA had significantly increased in the olfactory bulb, hippocampus, and cortex after 6 months of PM_2.5_ exposure (*p* < 0.05). We observed that 3 months of exposure to PM_2.5_ caused significantly higher expressions of t-tau and p-tau in the olfactory bulb (*p* < 0.05) but not in other brain regions. Beclin 1 was overexpressed in the hippocampus with 3 months of PM_2.5_ exposure, but significantly decreased in the cortex with 6 months exposure to PM_2.5_. Neuron numbers had decreased with caspase-3 activation in the cerebellum, hippocampus, and cortex after 6 months of PM_2.5_ exposure.

**Conclusions:**

Chronic exposure to low-level PM_2.5_ could accelerate the development of neurodegenerative pathologies in subjects with hypertension.

**Supplementary Information:**

The online version contains supplementary material available at 10.1186/s12989-020-00388-6.

## Background

Particulate air pollution has been linked to initiation of neurodegenerative diseases (NGDs) [1, 2]. Results from an aging study cohort indicated that exposure to traffic-related pollution and black carbon was associated with decreases in cognition function [3]. Other results from the Nurses’ Health Study Cognitive Cohort in the US showed that exposure to coarse (with an aerodynamic diameter of 2.5 ~ 10 μm) (PM_2.5–10_) and fine (PM_2.5_) particulate matter size fractions was associated with cognitive declines [4]. Exposure to traffic-related air pollution was associated with an increased risk of Parkinson’s disease (PD) [5]. An increased risk of hospital admission for dementia and PD was associated with an increase in PM_2.5_ as observed in 50 northeastern US cities [6]. These cohort data provide evidence that particulate matter (PM) may be a risk factor contributing to the development of NGDs; however, the underlying pathophysiology remains unclear.

Neuroinflammation is a critical mechanism in the development of NGDs. Our previous study indicated that oxidative stress and inflammation with short-term memory deficiencies occurred after 3 and 6 months of exposure to traffic-related PM_1_ (< 1 μm in aerodynamic diameter) in Sprague-Dawley rats [7]. Consistently, alterations in motor activity, spatial learning, and memory and emotional behaviors due to exposure to diesel exhaust particles (DEPs) were observed in vivo [8–10]. Acute exposure to DEPs at high concentrations (250 ~ 300 μg/m^3^) induced oxidative stress and neuroinflammation [8]. Costa and colleague indicated that neurogenesis injury in the hippocampus by DEPs was associated with activation of microglia. In vivo results provided evidence that DEPs caused oxidative stress, inflammation, and cell death in human neuroblastoma cells, which could be associated with regulating tau and autophagy expressions in the brain [11]. Physiological changes, inflammation, oxidative stress, and an unfolded protein response occurred after PM_2.5_ exposure, the responses of which differed with different size fractions of PM_2.5_ [12].

Deposition of insoluble proteins in cells of the neuromuscular system was characterized in NGDs. Clinically, tau protein accumulation is considered a biomarker of NGDs in clinical diagnoses [13, 14]. Overexpression of tau can cause its hyperphosphorylation [15], which leads to tau aggregation and the appearance of smaller proteolytic fragments [16]. Regulation of autophagy has a cleansing role in removing tau accumulations from cells, which is believed to be an important function in maintaining the brain’s health [16]. Previous studies observed that PM_2.5_ caused dysfunction in removing damaged proteins by autophagy [17, 18]. Also, DEPs induced neuroinflammation, oxidative stress, and neurodegenerative-related tau overexpression and regulation by autophagy in human IMR-32 neuroblastoma cells [11]. Autophagy dysfunction can lead to the initiation of NGDs such as Alzheimer’s disease (AD) [19]. However, chronic effects of PM_2.5_ on tau and autophagy expressions in the brain remain unclear.

During inhalation, PM is able to deposit in the upper airway such as head and nasal regions of humans [20]. For example, ultrafine particles (UFP; ≤100 nm) is mainly deposited by diffusion which makes it more likely to deposit on the olfactory epithelium. Additionally, UFP could translocate to brain via the olfactory epithelium [21, 22]. One study indicated significant hyperphosphorylated tau neurofibrillary tangles, vascular amyloid, neuronal amyloid accumulation, alpha-synuclein aggregates, and neurites in the olfactory bulbs after exposure to high levels of PM_2.5_ in 179 residents living in Metropolitan Mexico City [23]. Increasing reports suggest that olfactory deficits are associated with early evidence of AD pretangled subcortical and cortical hyperphosphorylated tau.

Cardiovascular diseases and metabolic syndrome are recognized as risk factors for the development of NGDs [24, 25]. A previous study showed that exposure to traffic-related air pollution impaired the brain’s microvascular integrity in a high-fat diet animal model [26]. However, a paucity of evidence is available for neurotoxicity caused by PM_2.5_ in hypertensive animals. We hypothesized that patient with hypertension is population-at-risk for development of neurodegenerative disease by chronic exposure of PM_2.5_. The objective of the present study was to investigate the effects of PM_2.5_ on neuropathophysiology in a hypertensive animal model. We examined behavioral alterations, lipid peroxidation, tau and autophagy expressions, neuronal death, and caspase-3 levels after chronic pulmonary exposure in spontaneously hypertensive (SH) rats.

## Results

### Characterization of urban PM_2.5_

**Fig. 1:**
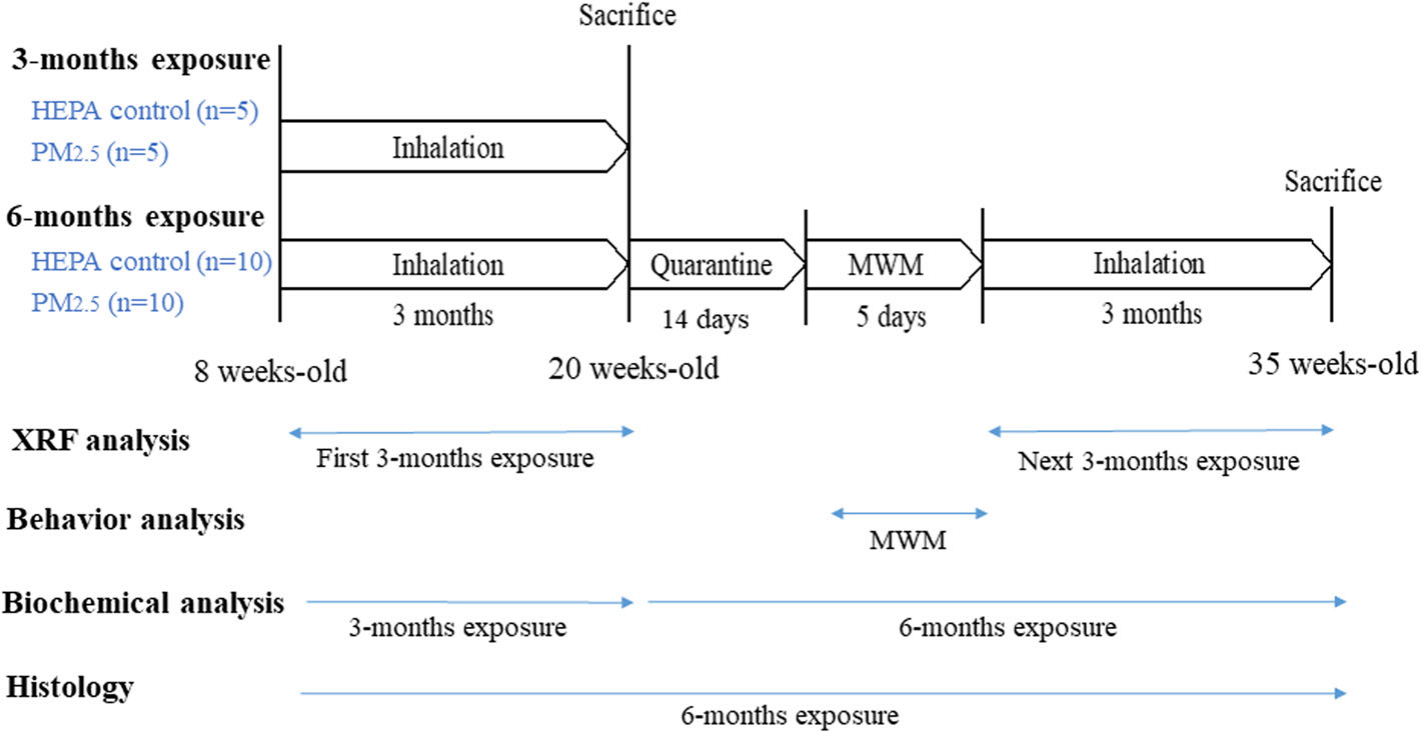
Overview of the experimental design for investigating the effects of fine particulate matter (PM_2.5_; PM with an aerodynamic diameter of < 2.5 μm) on neurotoxicity in spontaneously hypertensive (SH) rats. The whole bodies of 8-week-old SH rats in the high-efficiency particulate air (HEPA) and PM_2.5_ groups were exposed to urban PM_2.5_ for 3 and 6 months. In the 6-month group, a Morris water maze (MWM) was used to observe behavioral changes in SH rats after 3 months of exposure. After the MWM, rats were followed up for a subsequent 3 months of exposure to PM_2.5_. Rats were euthanized after 3 or 6 months of exposure for biochemical analyses. The 6-month group was histologically examined. Additionally, urban PM_2.5_ was collected onto Teflon filters for metal analyses

**Fig. 2:**
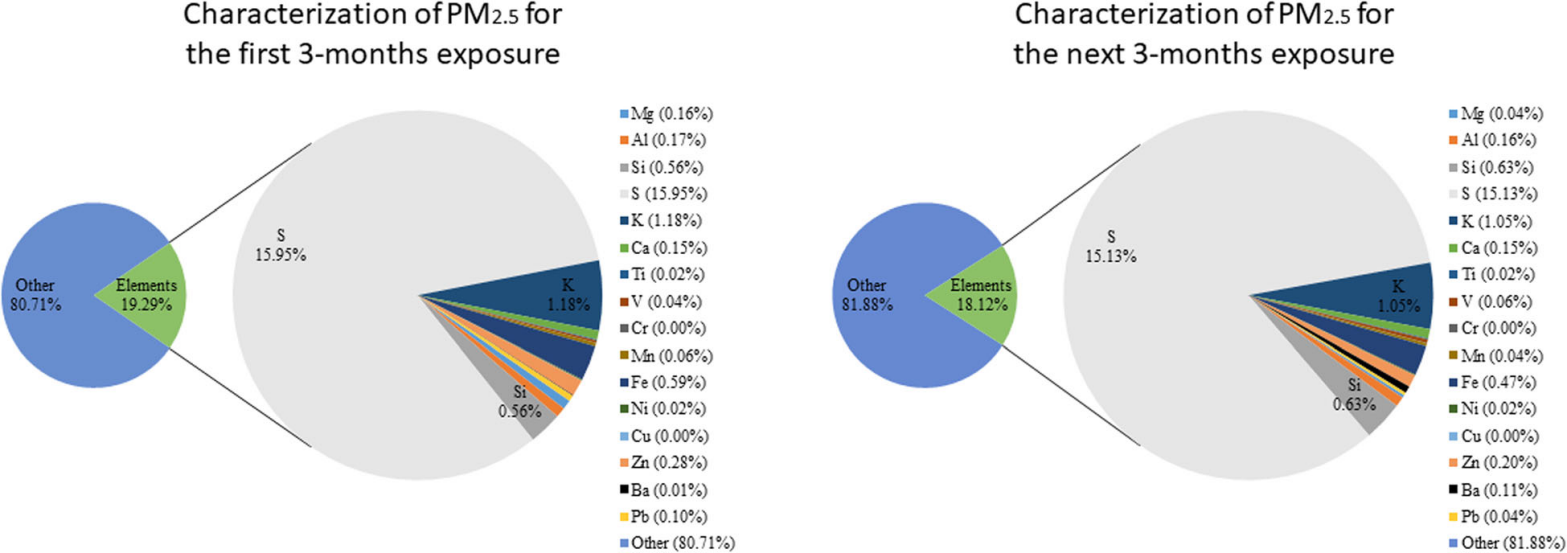
Characterization of elements in particulate matter with an aerodynamic diameter of < 2.5 μm (PM_2.5_) for an initial 3 months of exposure and a subsequent 3 months of exposure using energy dispersive x-ray fluorescence (ED-XRF) spectrometry. S, K, Si, and Fe were dominant in the first 3-month and subsequent 3-month PM_2.5_ samples

The experimental design is shown in Fig. 1. SH rats were exposed to either 3 or 6 months of urban PM_2.5_ during the study period. PM_2.5_ mass concentrations were 8.6 ± 2.5 and 10.8 ± 3.8 μg/m^3^ μg/m^3^ for the 3- and 6-month exposure periods, respectively. Meteorological conditions and gaseous pollution were referenced from the nearby EPA Guting air quality monitoring station during the study period (Table S1). The average temperature was 20.2 ± 5.0 °C and relative humidity was 80.8% ± 9.6%. Levels of NO_2_, SO_2_, and O_3_ exposed to the rats (HEPA and PM_2.5_ groups) were 21.6 ± 6.3, 2.6 ± 1.1, and 26.3 ± 10.7 ppb, respectively. We then determined elements of PM_2.5_ for the first 3 months and the subsequent 3 months of exposure (Fig. 2). We observed that elements in the first 3 months and subsequent 3 months of exposure respectively accounted for 19.29 and 18.12% of the total PM_2.5_. Consistently, S, K, Si, and Fe were dominant in the both first 3 months and subsequent 3 months of PM_2.5_.

### MWM

**Fig. 3:**
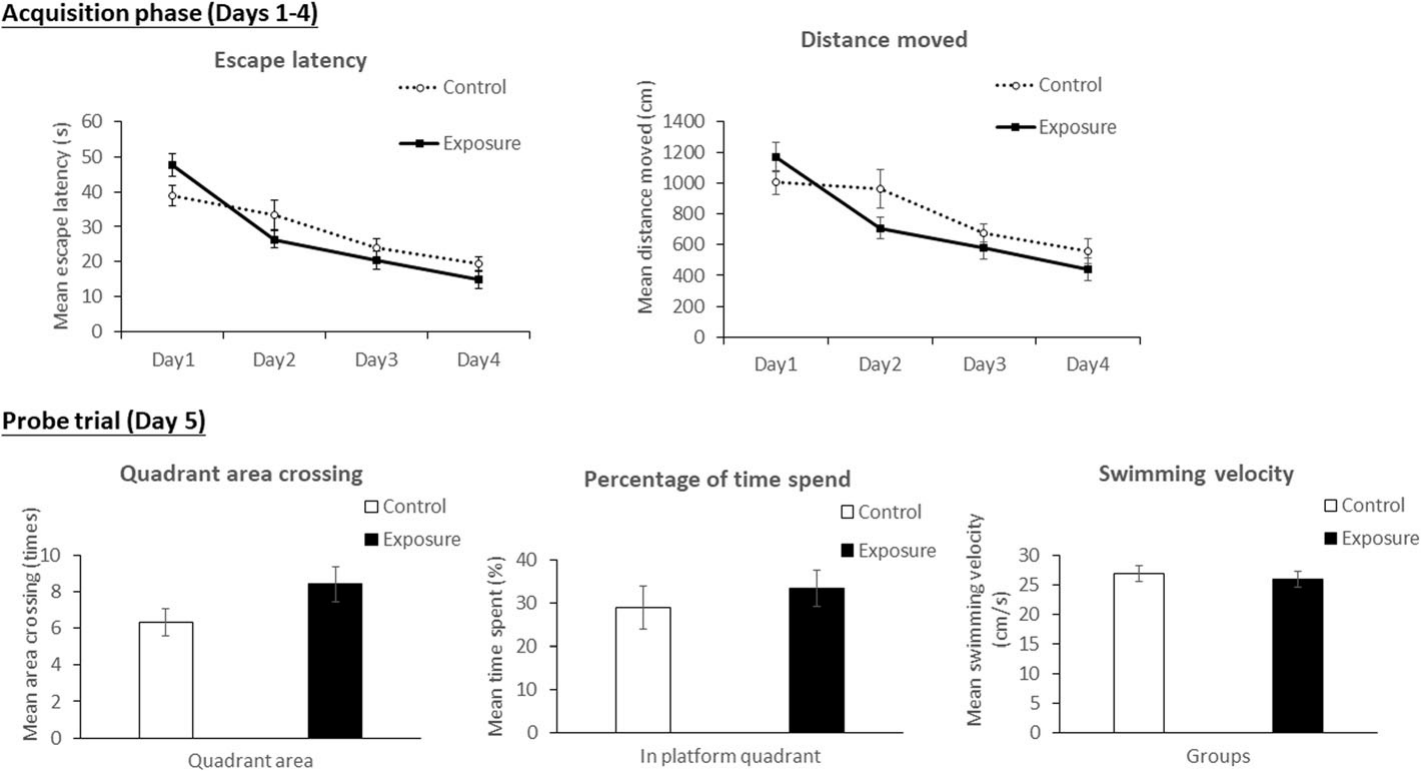
Morris water maze for examining spatial learning (acquisition phase, days 1 ~ 4) and memory ability (probe trial, day 5) in SH rats after 3 months of exposure to particulate matter with an aerodynamic diameter of < 2.5 μm (PM_2.5_; exposure group). There were no significant differences in the mean escape latency, mean distance moved, mean area crossing, mean time spent, or mean swimming velocity between the control (high-efficiency particulate air; HEPA) and exposure groups (PM_2.5_)

Figure 3 shows the acquisition phase (days 1 ~ 4) and probe trial (day 5) using the MWM to respectively examine spatial learning and memory ability. There were no significant differences in the escape latency or distance moved between the control and exposure groups. For the probe trial, there were no significant differences in the mean area crossing, mean time spent, or mean swimming velocity between the control and exposure groups.

### MDA

**Fig. 4:**
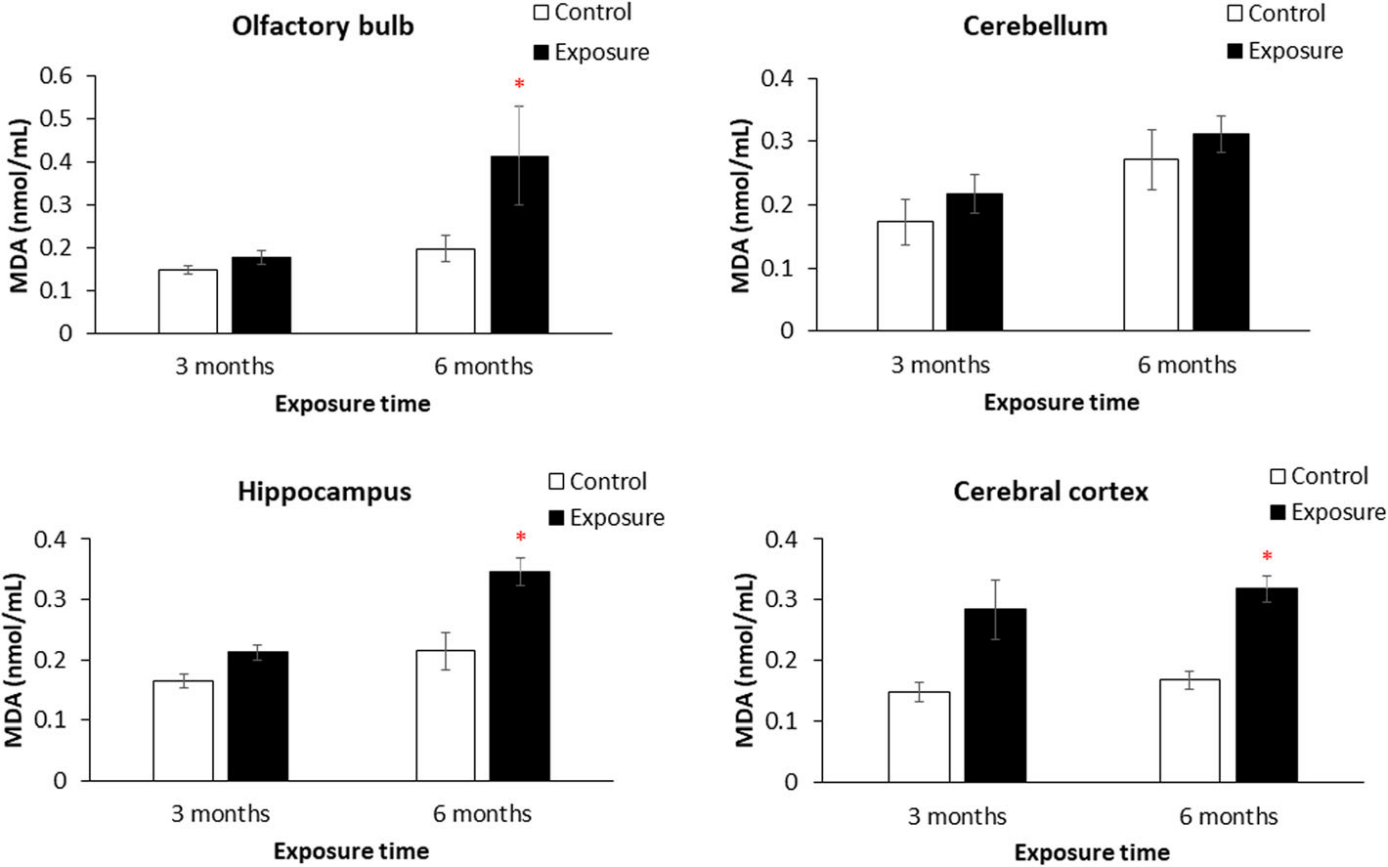
Levels of malondialdehyde (MDA) in the olfactory bulb, cerebellum, hippocampus, and cortex after 3 and 6 months of exposure to particulate matter with an aerodynamic diameter of < 2.5 μm (PM_2.5_; exposure group). Levels of MDA had significantly increased in the olfactory bulb, hippocampus, and cortex after 6 months of PM_2.5_ exposure compared to the control (high-efficiency particulate air; HEPA). * p < 0.05

Figure 4 shows MDA levels in the olfactory bulb, cerebellum, hippocampus, and cortex after 3 and 6 months of exposure. There was no significant difference of MDA between the control group and the group with 3 months of exposure. However, MDA levels were significantly higher in the olfactory bulb, hippocampus, and cortex after 6 months of exposure compared to the control group (*p* < 0.05).

### Tau expressions

**Fig. 5:**
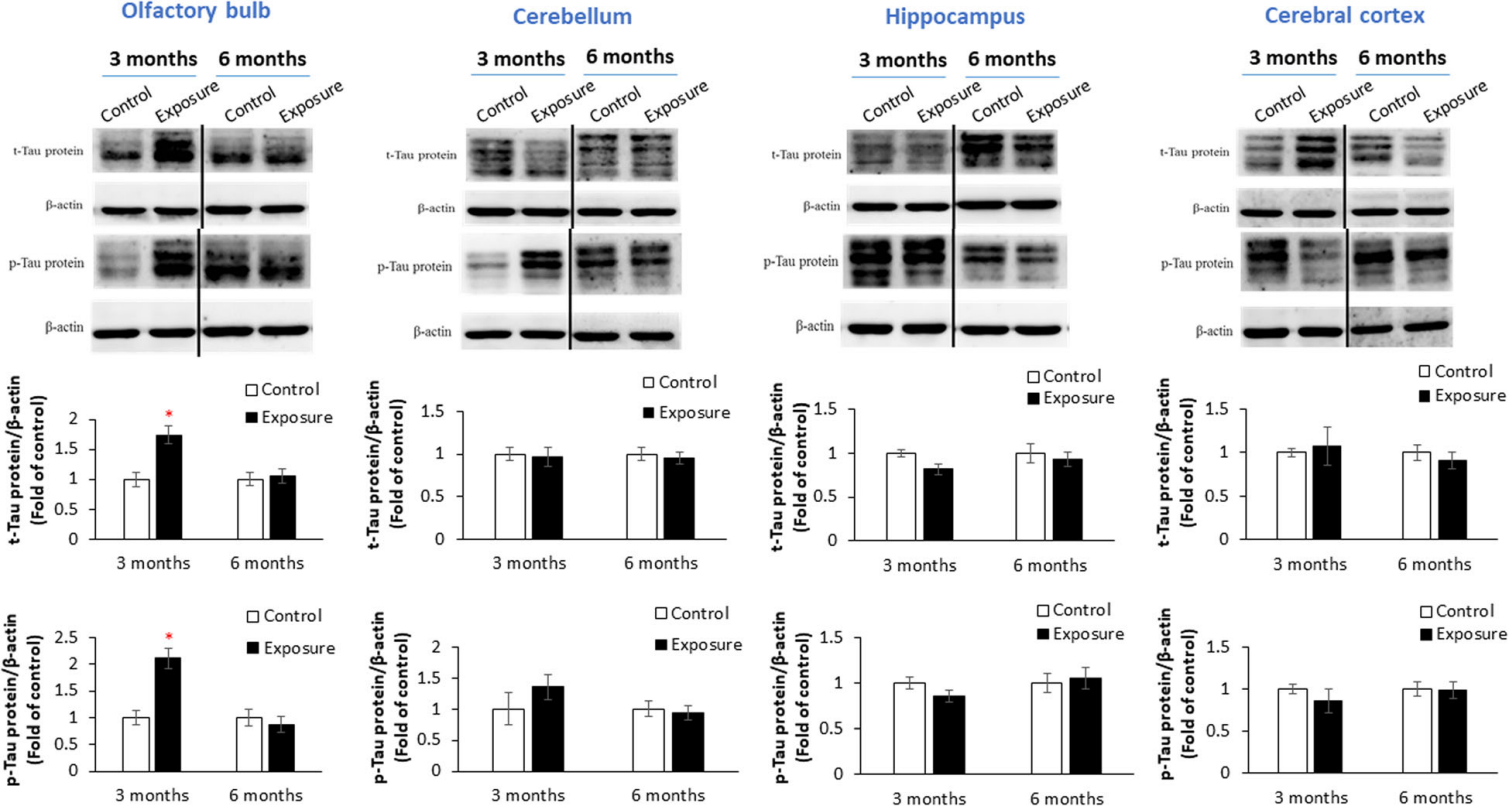
Expressions of total (t)-tau and phosphorylated (*p*)-tau in the olfactory bulb, cerebellum, hippocampus, and cortex after 3 and 6 months of exposure to particulate matter with an aerodynamic diameter of < 2.5 μm (PM_2.5_; exposure group). Three months of exposure to PM_2.5_ caused significant expressions of t-tau and p-tau in the olfactory bulb compared to the control (high-efficiency particulate air; HEPA). * p < 0.05

Figure 5 shows expressions of t-tau and p-tau in the olfactory bulb, cerebellum, hippocampus, and cortex after 3 and 6 months of exposure. We observed that 3 months of exposure to PM_2.5_ caused significant expressions of t-tau and p-tau in the olfactory bulb compared to the control (*p* < 0.05). However, there were no significant alterations in t-tau or p-tau in the cerebellum, hippocampus, or cortex after PM_2.5_ exposure. Consistently, we observed no significant difference in t-tau in the cerebellum, hippocampus, or cortex of the rat brain after 6 months of PM_2.5_ exposure based on IHC observations (Fig. S4).

### Beclin 1 expression

**Fig. 6:**
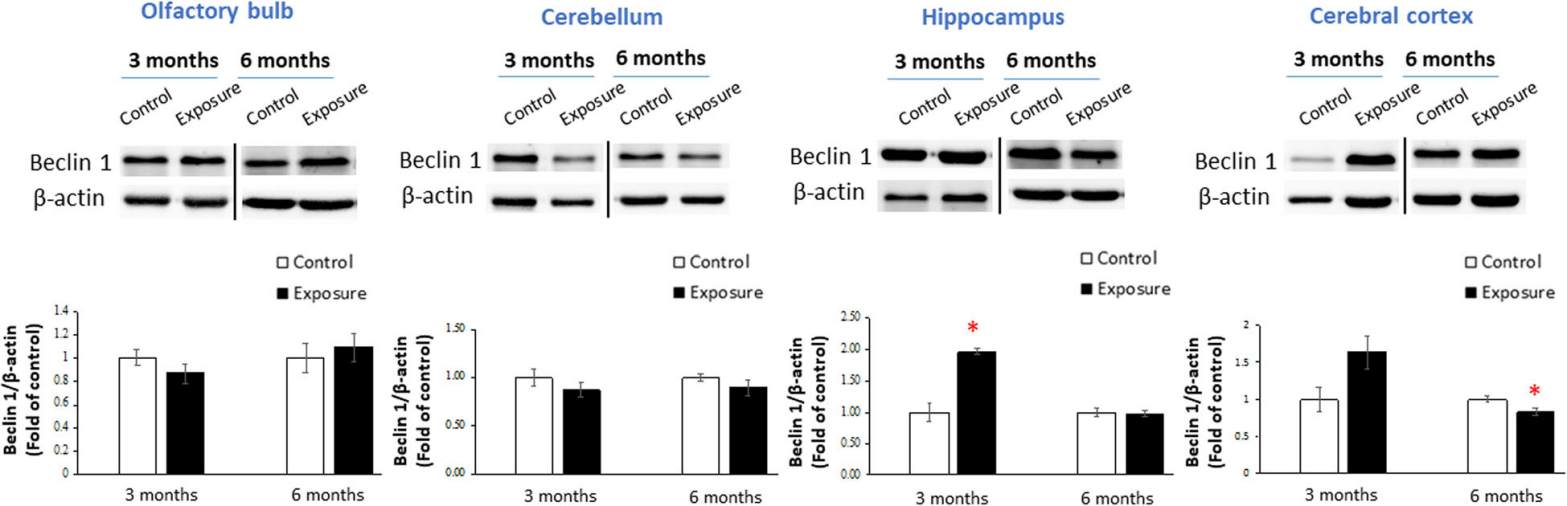
Expressions of beclin 1 in the olfactory bulb, cerebellum, hippocampus, and cortex after 3 and 6 months of exposure to particulate matter with an aerodynamic diameter of < 2.5 μm (PM_2.5_; exposure group). Three months of exposure to PM_2.5_ (exposure group) caused significant expression of beclin 1 in the hippocampus compared to the control (high-efficiency particulate air; HEPA), whereas 6 months of exposure of PM_2.5_ significantly decreased the expression of beclin 1 in the cortex compared to the control. * p < 0.05

Figure 6 shows expressions of beclin 1 in the olfactory bulb, cerebellum, hippocampus, and cortex after 3 and 6 months of exposure. We observed that 3 months of exposure to PM_2.5_ caused significant expression of beclin 1 in the hippocampus compared to the control (*p* < 0.05), whereas 6 months of exposure to PM_2.5_ caused a significant decrease in beclin 1 expression in the cortex compared to the control (*p* < 0.05).

### Neuron loss and caspase-3 activation

**Fig. 7:**
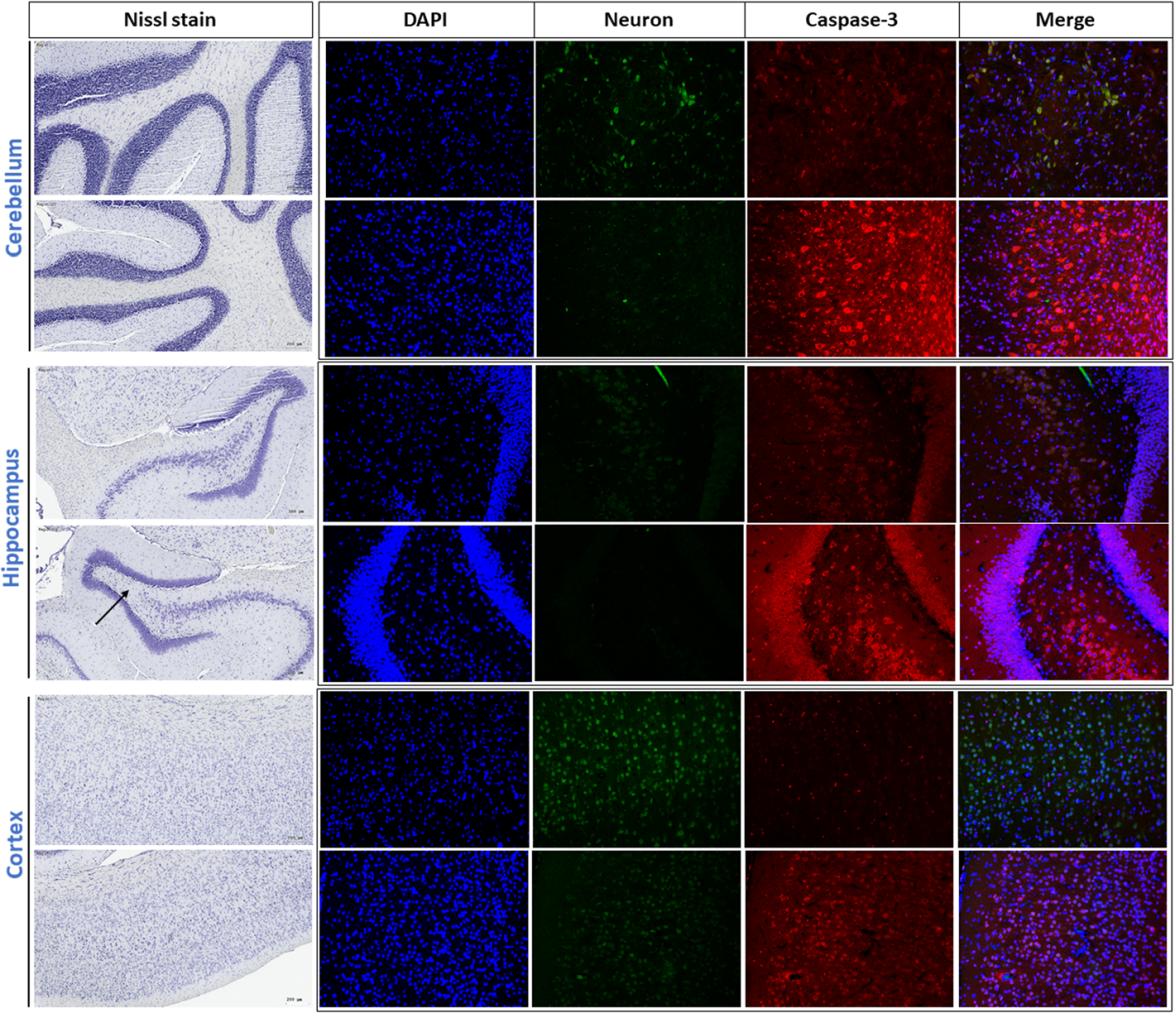
Neuron loss and activation of caspase-3 expression based on Nissl staining and IHC in the cerebellum, hippocampus, and cortex after 6 months of exposure to particulate matter with an aerodynamic diameter of < 2.5 μm (PM_2.5_; exposure group). For Nissl staining, the neuron number had significantly decreased in the hippocampus after 6 months of PM_2.5_ exposure (arrow). Neuron signals were also reduced in the cerebellum, hippocampus, and cortex after 6 months of PM_2.5_ exposure compared to the high-efficiency particulate air (HEPA) control. Caspase-3 signals were significantly activated in the cerebellum, hippocampus, and cortex by PM_2.5_ exposure compared to the control (HEPA). DAPI, blue; neurons, green; caspase-3, red. The scale bar for Nissl stain is 200 μm. The images for DAPI, neuron and caspase-3 were taken at 10x magnification

Figure 7 shows levels of neuron loss and caspase-3 activation based on Nissl staining and IHC in the cerebellum, hippocampus, and cortex after 6 months of exposure. First, the neuron number decreased in the hippocampus after 6 months of PM_2.5_ exposure (arrow). We further observed that neuron signals were also reduced in the cerebellum, hippocampus, and cortex after 6 months of PM_2.5_ exposure compared to the control (green color). Next, caspase-3 signals were activated in the cerebellum, hippocampus, and cortex by PM_2.5_ exposure compared to the control. However, we observed that few neuron cells were co-localized with caspase-3 activation based on merged images in the three brain regions. Results showed that caspase-3 was mainly activated in non-neuron cells.

### Histology

Figure S5 shows results of brain histology in the cerebellum, hippocampus, and cortex after 6 months of exposure to the HEPA control and PM_2.5_. No significant pathological changes were observed in the three regions of the brain after this exposure.

## Discussion

NGDs have been linked to air pollution based on epidemiological evidence. Hypertension is also recognized as a risk factor for NGDs [27, 28]. In the present study, we observed that pulmonary inflammatory infiltration occurred after 3- and 6-months of exposure to PM_2.5_ and/or HEPA without significant alterations in body weight (Figs. S2 and S3). Subpleural alveolar infiltration of mononuclear cells was observed in the lungs after 3- and 6-months exposure of HEPA and/or PM_2.5_ (Fig. S2). The observation suggests that the rats exposed to gaseous pollution and/or PM_2.5_ had adverse effects of histological changes of the lungs after exposure. The results could support the insignificant difference in certain biochemical results observed between PM_2.5_ and HEPA groups in this study. However, it is still unclear that neurotoxicity is occurred by PM_2.5_ or gaseous pollutants. In the present study, neurotoxicity was investigated after chronic inhalation of urban PM_2.5_ and/or gaseous pollution (HEPA) in SH rats. Four major findings are reported in the present study: (1) lipid peroxidation occurred in the olfactory bulb, hippocampus, and cortex after 6 months of exposure to PM_2.5_; (2) tau proteins (total and phosphorylated) were upregulated in the olfactory bulb by 3 months of exposure to PM_2.5_; (3) alterations in beclin 1 expression were observed in the hippocampus and cerebellum due to PM_2.5_ exposure; and (4) neuron loss and caspase-3 activation were induced by 6 months of exposure to PM_2.5_. Therefore, PM_2.5_ could accelerate the development of neurodegenerative pathologies in hypertension subjects.

Air pollution may be a risk factor for NGDs. The US Environmental Protection Agency (EPA) estimates that more than 100 million people live in areas that exceed the recommended US EPA air quality levels (Office of Air Quality Planning and Standards and United States, EPA, Air Quality Trends Analysis Group). Notably, chronic exposure to traffic-related PM was reported to increase the risk of NGDs in the US [29] and Canada [30]. In Taiwan, a similar association was identified based on data analyzed from a national population-based cohort study [31]. They showed that 10 years of exposure to PM_2.5_ and ozone were associated with an increased incidence of AD. To study possible underlying mechanisms in the brain after PM_2.5_ exposure, a traffic-dominated urban region in Taipei, Taiwan was selected for the experimental site in the present study. The temperature, RH, NOx, SO_2_, and O_3_ were referenced from the nearby Guting air quality monitoring station during the study period. Levels of NO_2_, SO_2_, and O_3_ to which rats were exposed were slightly higher than those in our previous study [7]. For PM_2.5_ exposure, rats were continually exposed to 8.6 and 10.8 μg/m^3^ PM_2.5_ for 3 and 6 months, respectively. Average PM_2.5_ levels in both exposure periods (3 and 6 months) were relatively lower than World Health Organization (WHO) PM_2.5_ guidelines (25 μg/m^3^ for the 24-h average) [32]. We further investigated elements in PM_2.5_, and S, K, Si, and Fe were mainly observed in PM_2.5_ during the exposure periods. The dominant elements suggest secondary aerosols (e.g., S), soil dust (e.g., Si), and metal/traffic (e.g., Fe) were the main emission sources in the study area [33]. Some elements, such as S and Fe, are recognized as a source for oxygen reactive species (ROS) that are able to cause cellular oxidative damage after inhalation [34, 35]. Increasing evidence indicates that exposure to metal-containing PM_2.5_ causes neurotoxicity based on human [36] and animal reports [37]. However, effects of pulmonary exposure to low-levels of PM_2.5_ on neurotoxicity remains unclear.

Chronic exposure to air pollution was observed to change behavior in animals [7] and their offspring [38]. However, the effect of PM_2.5_ on behavior changes in hypertension is still poorly understood. In the present study, SH rats, an animal model with essential/primary hypertension, was used to study the contributions of PM_2.5_ on neurotoxicity in hypertension. SH rats is commonly used to study interactions between air pollution and cardiovascular diseases [39–41]. Firstly, we used the MWM to examine spatial learning, according to a previous report [42]. There was no significant difference in spatial learning between the control and exposure groups. Previous reports showed that air pollution exposure caused in vivo behavioral alterations, such as anxiety [43] and short-term memory [7, 44]. Contrarily, another study observed no significant changes in in vivo behaviors after air pollution exposure [45]. Previous studies indicated that hypertension is linked to NGDs [27, 28], leading to memory deficiencies [46, 47]. It is worthy to note that stress could be occurred by behavior examination in rats, affecting pathology change and other biological responses in brain [48]. However, the rats were euthanized after 3 months of MWM in the present study. Thus, the effects of behavior testing on the biochemical results could be insignificant. Together, the insignificant alteration in behavior after air pollution exposure observed in the present study could have resulted from the animal model we used.

Lipid plays an important role of NGDs due to its high concentration in central nervous system, which involved in many central nervous system disorders and injuries that involve deregulated metabolism [49]. Transition metals such as Fe identified in PM_2.5_ are capable of redox cycling [50] and generate superoxide and hydroxyl radicals [51], through a direct mechanism, the Fenton reaction [34, 52, 53]. When ROS interact with lipids, lipids are degraded by ROS thereby producing lipid peroxidation. Lipid peroxidation is able to disrupt membrane organization and cause the functional loss/modification of proteins and DNA [54]. After 6 months of exposure to PM_2.5_, we observed significantly increased lipid peroxidation (as determined by MDA) in the olfactory bulb, hippocampus, and cortex of SH rats. Previous reports consistently indicated that PM_2.5_ exposure in the lungs caused significant lipid peroxidation in different brain regions in vivo [55, 56]. Particulate air pollution is able to cause barrier damage such as nasal epithelium [57], leading to integrity disruption. Olfactory epithelium has recently recognized to be an important target to interact with particulate matter. Oxidative stress and infalammation was occurred by particles between olfactory epithelium and brain [58]. But the responses may delayed depends on proximal and distal brain regions [59]. Repeated injury of these barriers is able to impair their integrity, leading to PM crossing the barriers and entering the brain. Also, oxidative stress is able to directly alter amyloid-β production. The ROS-occurred production of lipid peroxidation may trigger generation of toxic amyloid-β42 species in the brain [60]. Clinically, lipid peroxidation is used as an indicator of ROS that cause NGDs [61]. Together, chronic exposure of PM_2.5_ causes formation of lipid peroxidation, which may increase the risk of NGDs.

Cellular deposition of insoluble proteins in the neuromuscular system was linked to neurological disease. Tau, for example, was observed to accumulate in intraneurons, which was suggested to be a biomarker of the development of AD [14]. Generally, olfactory sensory neurons are dedicated to processing odor information. Currently, the olfactory system is recognized to decrease in function due to physiological changes during normal aging [62] and NGDs [63]. Olfactory sensory neurons are associated with the development of AD [64–66]. In our study, we observed t-tau and p-tau overexpression in the olfactory bulb after 3 months of PM_2.5_ exposure in SH rats. A report indicated that activation of microglia caused the increase of p-tau aggregates in the olfactory system [67]. The results from Mexico City study showed that olfactory bulbs is the targets for air pollution, which caused p-tau in olfactory bulb of young city residents [68]. A study showed that the olfactory bulb is nearly equally vulnerable to tau and α-synuclein pathologies in AD with amygdala Lewy bodies [65]. Together, olfactory bulb could be the first target for sub-chronic PM_2.5_ exposure, leading to p-tau aggregates in SH rats. However, the alteration in t-tau and p-tau of olfactory bulb was not observed by 6-months exposure of PM_2.5_. The insignificant results could be resulted from the effects of gaseous pollution after chronic exposure in rats. Taken together, PM_2.5_ exposure causing overexpression of t-tau and p-tau could be associated with the early development of neurotoxicity.

Autophagy is a cleansing mechanism to remove accumulations of tau proteins from the brain. The Bcl-2-interacting protein, beclin 1 (Atg6), and the microtubule-associated protein-1 light chain 3B (LC3B, Atg8) are considered to be major regulators of autophagy [69]. In the present study, we observed that beclin 1 was overexpressed in the hippocampus after 3 months of PM_2.5_ exposure in SH rats. This observation is in line with our previous report that chronic exposure to traffic-related air pollution activated autophagy expression in the SD rat hippocampus [7]. Additionally, LC3II (conjugated LC3) was significantly upregulated by carbon black and diesel exhaust particles (DEPs), particularly DEPs, in microglial BV2 cells [70]. Autophagy activation could be associated with insignificant alterations in t-tau and/or p-tau of brains of SH rats after exposure. However, we observed downregulated autophagy expression in the cortex after 6 months of PM_2.5_ exposure in SH rats. Our previous study showed that DEPs did not activate LC3II expression in neuroblastoma cells [11]. The difference could have resulted from cell types distributed in different brain regions or autophagy dysfunction occurring due to chronic PM_2.5_ exposure.

Notably, we observed neuron loss in the hippocampus of SH rats after 6 months of exposure to PM_2.5_. We next investigated expressions of neuronal cells in different brain regions. Our observations suggested that neuron cells had decreased in the cerebellum, hippocampus, and cortex after 6 months of exposure to PM_2.5_. Consistently, our previous study showed that DEPs caused the cell death of BV2 neuroblastomas [11], which may have been related to oxidative stress in the brain after exposure as determined by lipid peroxidation in our current results. Next, we observed that caspase-3 expression occurred in the cerebellum, hippocampus, and cortex after 6 months of exposure to PM_2.5_. However, caspase-3 expression was co-localized with either neuron cells or other cells in the three brain regions in the present study. Another study indicated that microglial activation occurred due to neuroinflammation by DEPs [70], leading to neuronal cell death [71]. Therefore, it is reasonable to suspect that chronic exposure to PM_2.5_ can cause the death of neurons and brain cells. Further studies are required to understand interactions between PM_2.5_ and brain cells and its underlying mechanisms.

We observed lung infiltration after chronic PM_2.5_ exposure in SH rats, but there were no significant pathological changes in brains of SH rats. A few limitations of this study should be noted. We examined elements of PM_2.5_ exposure; however, we did not examine the organic fractions of PM_2.5_. Also, oral uptake of PM_2.5_ in the rats was not observed. Rats were euthanized using CO_2_ at each time point, which could induce stress that affect the results. A normotensive and a clean air controls may be required to understand the effects of PM_2.5_ on the development of NGDs in the hypertensive rat model. In the present study, we did not observe significant behavioral alterations due to PM_2.5_ exposure in the model. More behavior experiments should be performed in the future works. Other models could have been used, such as aging rats, to examine the role of PM_2.5_ in NGDs.

## Conclusions

In conclusion, neuropathological change was occurred after chronic exposure to low-level PM_2.5_ in SH rats. Lipid peroxidation, neuron loss, and caspase-3 activation were identified in brains after 6 months of exposure to PM_2.5_. Tau and beclin 1 overexpressions were caused by 3 months of exposure to PM_2.5_. Notably, the olfactory bulb may be an important target organ by PM_2.5_. Our results showed that chronic exposure to low-level PM_2.5_ could accelerate the development of neurodegenerative pathology in subjects with hypertension.

## Materials and methods

### Animals

Seven-week-old male SH rats obtained from the National Laboratory Animal Center (Taipei, Taiwan) were used in this study. Rats were housed in plastic cages under a 22 ± 2 °C temperature, 55% ± 10% relative humidity, and a 12/12-h light/dark cycle. Lab Diet 5001 (PMI Nutrition International, USA) and water were provided ad libitum. All animal experiments complied with protocols of the Institutional Animal Care and Use Committee (IACUC) of National Taiwan University (Taipei, Taiwan; approval no. 20130531).

### Experimental design

The experimental design is shown in Fig. 1. SH rats in the high-efficiency particulate air (HEPA) and PM_2.5_ groups were whole-body exposed to urban air pollution for 3 and 6 months between November 2015 and May 2016 in Taipei City, Taiwan. For the 3-month group, rats were immediately decapitation. For the 6-month group, rats were quarantined after 3 months of exposure. Rats were then forced to perform a Morris water maze (MWM) for 5 days. After the MWM, rats were exposed to urban air pollution for the next 3 months. After being euthanized, an animal necropsy was performed, and tissues were collected as described previously [72]. In this study, all rats were euthanized using CO_2_ at each time point. For 3-month group, there were 5 rats used for biochemical analyses. For 6-month group, there were 6 rats used for biochemical analyses and 4 rats used for pathological observation. The olfactory bulb, cerebellum, cortex, and hippocampus of each rat were obtained and stored at − 80 °C. Also, brain samples were quickly removed from the skull and immersed overnight in 4% formaldehyde at 4 °C for pathological observation. Additionally, urban PM_2.5_ was collected onto Teflon filters for mass calculation and metal analyses.

### Whole-body exposure to urban PM_2.5_

SH rats were randomly assigned to two groups: (1) a HEPA-filtered air control group (exposed to gaseous pollution only), and (2) a PM_2.5_ group (exposed to PM_2.5_ and gaseous pollution). The whole-body exposure system used for rodent PM_2.5_ exposure was previously described [7, 73], and was located in an urban region with traffic-dominated emission sources (Taipei, Taiwan). Briefly, unconcentrated ambient air was introduced into the whole-body exposure system for 3 and 6 months. Our previous report showed that the coarse (PM_2.5–10_) and fine (PM_2.5_) size fractions of PM mass concentration respectively accounted for 0.4 and 99.6% of the whole-body exposure system [73]. Thus, rats were mainly exposed to PM_2.5_ in this study. Simultaneously, PM was collected onto Teflon filter substrates during the study period (7 days for an interval). Mass concentrations of PM_2.5_ were obtain from the filters followed by metal analyses. Meteorological conditions and gaseous pollution were referenced from the nearby EPA Guting air quality monitoring station during the study period.

### Elemental analysis

Energy dispersive x-ray fluorescence (ED-XRF) spectrometry (on a PANalytical Epsilon 5 ED-XRF analyzer, PANalytical, the Netherlands) was used to determine elemental concentrations in PM_2.5_ collected on Teflon filters. The ED-XRF procedure was described in a previous report [74]. Mg, Al, Si, S, K, Ca, Ti, V, Cr, Mn, Fe, Ni, Cu, Zn, Ba, and Pb were determined from material collected on Teflon filters. Metal concentrations are presented as a weight percentage of PM_2.5_ (%).

### Morris water maze (MWM)

The MWM was used to examine spatial learning, according to our previous report [42]. Briefly, the MWM consisted of a circular pool with a diameter of 120 cm and a height of 60 cm. A white platform (9 cm in diameter with a rough surface) was submerged 1 cm below the water surface. The pool was filled with water, and the water temperature was kept at 25 °C. The pool was divided into four equal quadrants, and each quadrant was marked with a different visual cue. There was two phases during the study period: an acquisition phase (days 1 ~ 4) and a probe trial phase (day 5) to respectively examine spatial learning and memory abilities. Each trial began by placing a rat in the pool, facing the wall of the tank opposite the midpoint of each quadrant. The sequence of the starting quadrant was randomly assigned. Once the rat had found the platform, it was allowed to stay there for 15 s. If a rat did not find the platform within 60 s, it was gently place on the platform by the experimenter and was allowed to stay there for 15 s. All data were recorded with a video camera-based system (Noldus Etho Vision 3.1, Wageningen, the Netherlands).

### Malondialdehyde (MDA) analysis

MDA was extracted from the olfactory bulb, cerebellum, cortex, and hippocampus according to a previously described method with minor modifications [75]. Details of the preparation and liquid chromatography-tandem mass spectrometry (LC-MS/MS) analysis are given in Supplymentary Information. Briefly, samples were reacted with 2,4-dinitrophenylhydrazine (DNPH) to derivatize the MDA-DNPH, followed by the LC-MS/MS analysis. MDA-DNPH was separated from other interfering substances on an LC system containing a Thermo Scientific autosampler and a Thermo Scientific Accela LC system with a 1250 quaternary pump (San Joes, CA, USA). A 15-cm (4.6 mm I.D) Syncronis C18 column was packed with 5 μm particles (Thermo Fisher Scientific, Waltham, MA, USA). A Thermo TSQ Quantum Access with a triple-quadrupole mass analyzer was applied to quantitate MDA-DNPH. MS/MS was carried out in selected reaction monitoring (SRM) mode with 1.5 mTorr of argon gas as the collision-induced dissociation (CID) gas. The LC-MS/MS system and data analysis were controlled and processed with Xcalibur software (Thermo Fisher Scientific). Method validation in terms of the linearity, and limits of detection (LODs) and quantification (LOQs), and the matrix effect were performed based on previous research and guidance from US Food and Drug Administration [76, 77]. Since an isotope-labeled internal standard of MDA-DNPH was unavailable, a matrix-matched calibration curve of MDA-DNPH was used to compensate for the matrix effect in SH rat brain samples, and LODs and LOQs were also determined in brain tissues from the control group of SH rats. Spectra of the LC-MS/MS analyses are given in Fig. S1.

### Western blot analysis

The Western blot analysis was described previously [7]. Briefly, total tau (t-tau; 1:1000), phosphorylated-tau (p-tau; 1:1000), beclin 1 (1:1000), and β-actin (1:1000) obtained from Cell Signaling (Danvers, MA, US) were immunoreactived with specific proteins. Quantitative data were obtained using Image-Pro vers. 4 (Media Cybernetics, Inc., MD, USA) for Windows. All data were adjusted to the control (multiples of change of the control).

### Immunohistochemistry (IHC) of brain tissues

Fixed brain samples were incubated with polyclonal antibodies against t-tau, whereas incubation with phosphate-buffered saline (PBS) served as a negative control. DAPI was used for nuclear staining. Microphotographs were acquired using a Motic Pathology slide scanner (Meyer Instrument, Houston, TX, USA). Also, brain sections were stained with NeuN (GeneTex, San Antonio, TX, USA), the cell apoptosis marker of activated caspase-3 (Cell Signaling Technology, Beverly, MA, USA), and nuclear-staining DAPI (Sigma-Aldrich, St. Louis, MO, USA). For neuron detection, a Nissl staining kit (MDS Analytical Technologies, Sunnyvale, CA, USA) was used to measure Nissl bodies in the cytoplasm of neurons. A fluorescent microscope (EVOS FL imaging system; Thermo Fisher Scientific) was used to obtain NeuN, caspase-3, and DAPI images. Automated microscopy (Tissuefaxs; TissueGnostics, Vienna, Austria) was used to obtain Nissl-stained images. The regions of interest were selected (*n* = 3 sections per region) based on significant changes and our previous reports [7, 70]. All images were taken under the same exposure time.

### Histology

Brain and lung sections were fixed, embedded in paraffin, and sectioned followed by staining with hematoxylin and eosin (H&E). Images were acquired using the Motic Pathology slide scanner. All histological examinations were conducted under light microscopy by a single histopathologist.

### Statistical analysis

Data are presented as the mean ± standard deviation (SD). The Wilcoxon rank sum test was used for comparisons between groups. Statistical analyses were performed using SAS 9.2 for Microsoft Windows (SAS Institute, Cary, NC, USA). The level of significance was set to *p* < 0.05.

## Supplementary Information

**Additional file 1: Table S1.** Meteorological and gaseous data measured by the EPA Guting air quality monitoring stations during the study period. **Figure S1.** Spectrums of MDA analysed by LC-MS/MS. **Figure S2.** Effects (3- and 6-months exposure) of PM2.5 on the inflammatory infiltration of lungs in SH rats. Subpleural alveolar infiltration of mononuclear cells was observed in the lungs after 3- and 6-months exposure of HEPA and/or PM2.5. **Figure S3.** Alteration in body weight of SH rats between HEPA (control) and PM2.5 (exposure) groups after 6-months exposure. **Figure S4.** IHC images of total Tau (t-Tau) in the cerebellum, hippocampus, and cortex of SH rats after 6 months of exposure to HEPA (control) and PM2.5 (exposure). Scar bar is 50 μm. **Figure S5.** Chronic effects (6-months exposure) of PM2.5 on the histological changes of cerebellum, hippocampus, and cortex in SH rats. Scale bar: 200 μm.
