# Supplementary material for: Neuropathology changed by 3- and 6-months low-level PM2.5 inhalation exposure in spontaneously hypertensive rats
Source: Part Fibre Toxicol. 2020 Nov 26;17:59. doi: 10.1186/s12989-020-00388-6 (PMC7691081; doi:10.1186/s12989-020-00388-6)
Supplement: Supplementary file 1 — Additional file 1: Table S1. Meteorological and gaseous data measured by the EPA Guting air quality monitoring stations during the study period. Figure S1. Spectrums of MDA analysed by LC-MS/MS. Figure S2. Effects (3- and 6-months exposure) of PM2.5 on the inflammatory infiltration of lungs in SH rats. Subpleural alveolar infiltration of mononuclear cells was observed in the lungs after 3- and 6-months exposure of HEPA and/or PM2.5. Figure S3. Alteration in body weight of SH rats between HEPA (control) and PM2.5 (exposure) groups after 6-months exposure. Figure S4. IHC images of total Tau (t-Tau) in the cerebellum, hippocampus, and cortex of SH rats after 6 months of exposure to HEPA (control) and PM2.5 (exposure). Scar bar is 50 μm. Figure S5. Chronic effects (6-months exposure) of PM2.5 on the histological changes of cerebellum, hippocampus, and cortex in SH rats. Scale bar: 200 μm. [file 12989_2020_388_MOESM1_ESM.pdf]

Supplementary Information

Table S1. Meteorological and gaseous data measured by the EPA Guting air quality monitoring stations during the study period

Meteorological and gaseous data (unit)	Mean ± SD (Min~Max)
**Temperature (°C)**	20.2 ± 5.0 (5.4~31.1)
**Relative humidity (%)**	80.8 ± 9.6 (54.9~96.1)
**NO_2 (ppb)**	21.6 ± 6.3 (10.0~50.0)
**SO_2 (ppb)**	2.6 ± 1.1 (0.9~8.3)
**O_3 (ppb)**	26.3 ± 10.7 (2.0~84.3)

NO_2: nitrogen dioxides; SO_2: sulfur dioxide; O_3: ozone.

Supplementary Information

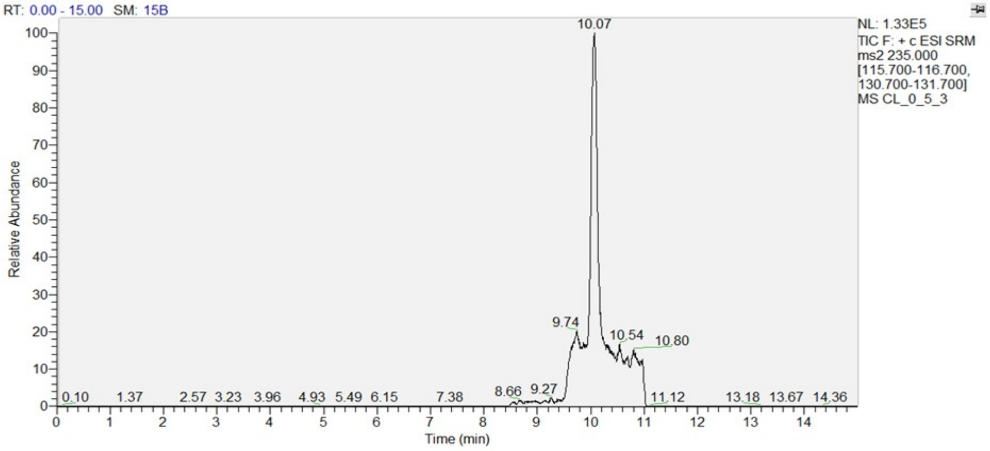

Figure S1. Spectrums of MDA analysed by LC-MS/MS.

Supplementary Information

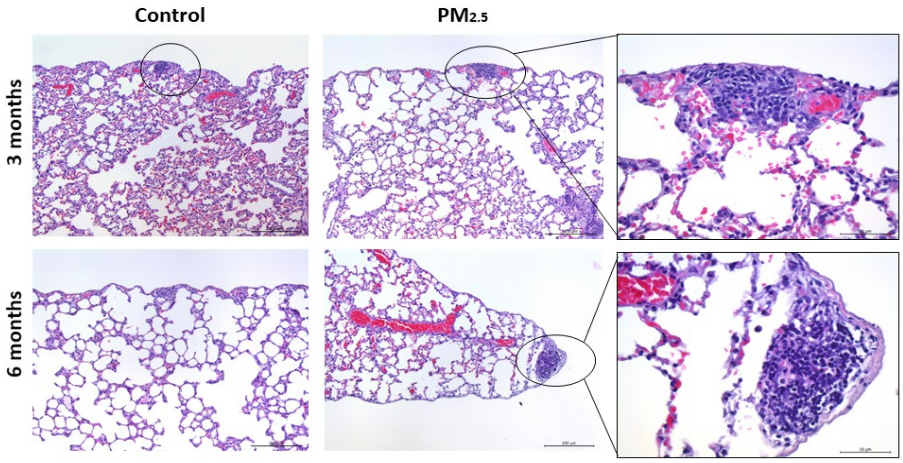

Figure S2. Effects (3- and 6-months exposure) of $PM_{2.5}$ on the inflammatory infiltration of lungs in SH rats. Subpleural alveolar infiltration of mononuclear cells was observed in the lungs after 3- and 6-months exposure of HEPA and/or $PM_{2.5}$.

Supplementary Information

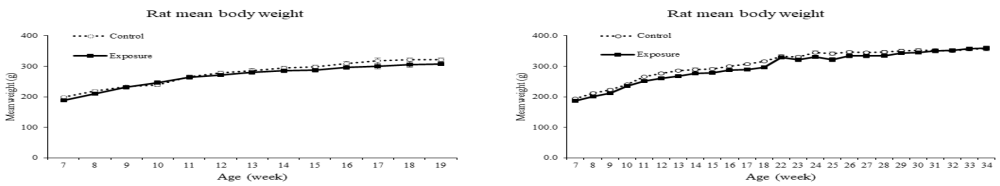

Figure S3. Alteration in body weight of SH rats between HEPA (control) and $PM_{2.5}$ (exposure) groups after 6-months exposure.

Supplementary Information

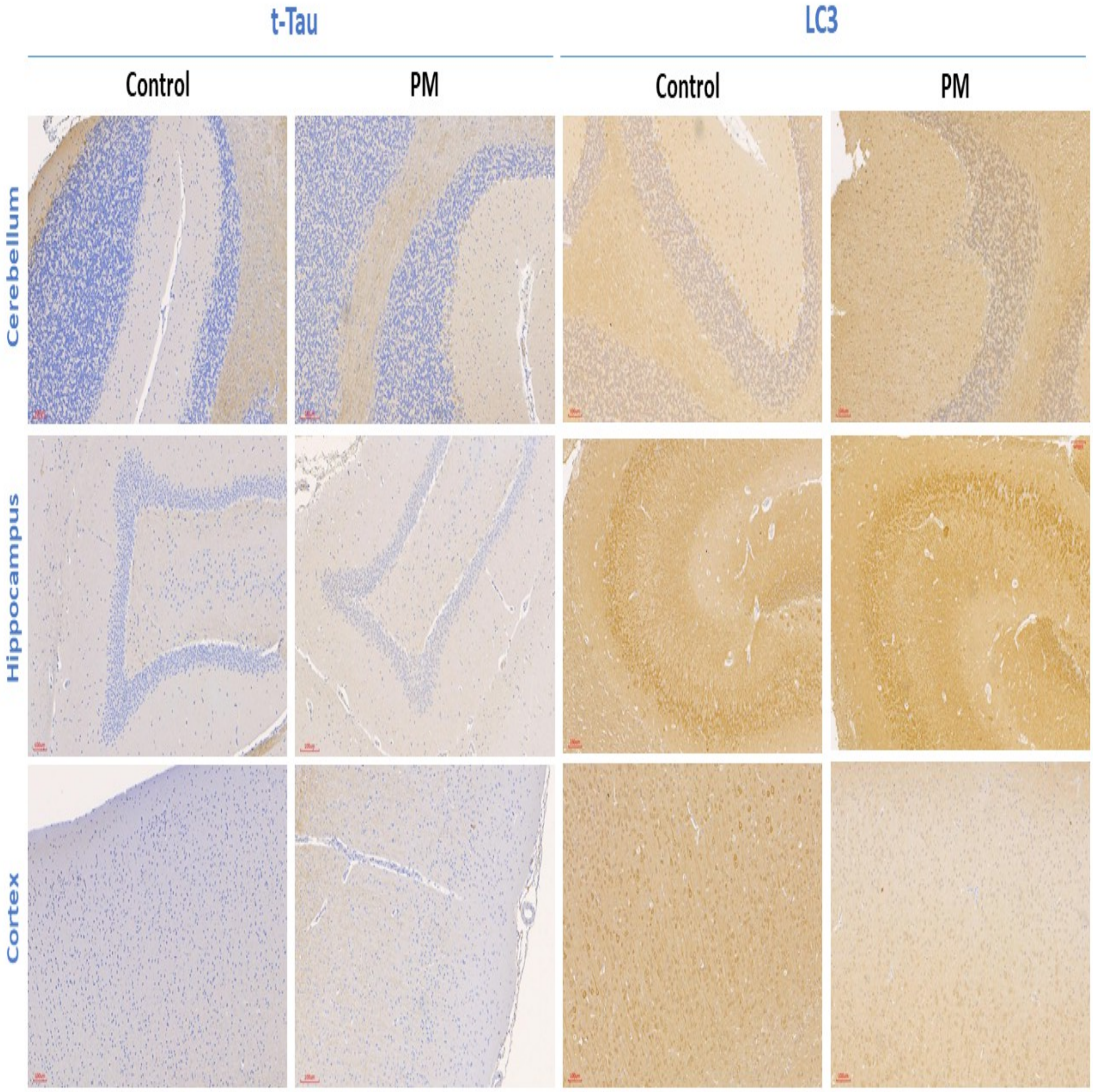

Figure S4. IHC images of total Tau (t-Tau) in the cerebellum, hippocampus, and cortex of SH rats after 6 months of exposure to HEPA (control) and $PM_{2.5}$ (exposure). Scar bar is 50 μm.

Supplementary Information

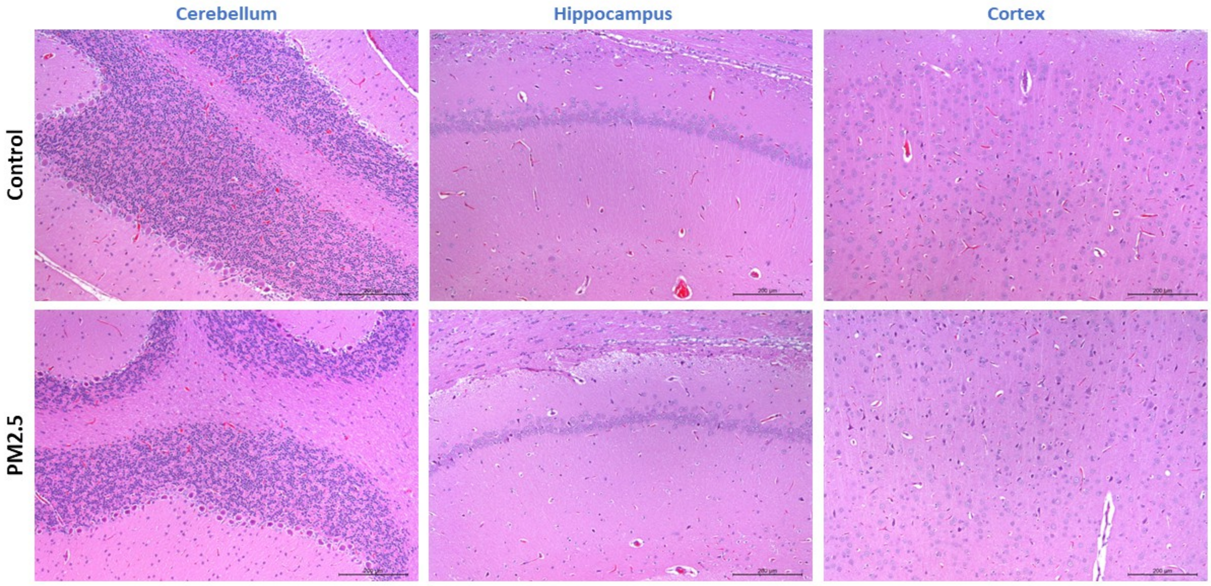

Figure S5. Chronic effects (6-months exposure) of $PM_{2.5}$ on the histological changes of cerebellum, hippocampus, and cortex in SH rats. scale bar: 200 μm
